# Millisecond Spin Relaxation Times of Distinct Electron and Hole Subensembles in MA_*x*_FA_1−*x*_PbI_3_ Perovskite Crystals

**DOI:** 10.1002/advs.76770

**Published:** 2026-07-28

**Authors:** Rongrong Hu, Sergey R. Meliakov, Dmitri R. Yakovlev, Bekir Turedi, Maksym V. Kovalenko, Manfred Bayer, Vasilii V. Belykh

**Affiliations:** ^1^ Experimentelle Physik 2 Technische Universität Dortmund Dortmund Germany; ^2^ School of Science Shanghai Institute of Technology Shanghai China; ^3^ Laboratory of Inorganic Chemistry, Department of Chemistry and Applied Biosciences ETH Zürich Zürich Switzerland; ^4^ Laboratory for Thin Films and Photovoltaics Empa‐Swiss Federal Laboratories for Materials Science and Technology Dübendorf Switzerland; ^5^ Research Center FEMS Technische Universität Dortmund Dortmund Germany

**Keywords:** longitudinal spin relaxation, perovskite single crystal, spin dynamics

## Abstract

The unique combination of outstanding optical quality and attractive spin properties opens new avenues for optical spin control in hybrid organic‐inorganic perovskite semiconductors. Using the optically detected magnetic resonance technique, we study the spins of electrons and holes in mixed‐cation ${\mathrm{MA}}_{x}{\mathrm{FA}}_{1-x}{\mathrm{PbI}}_{3}$ single crystals with x=0.4 and 0.8. Multiple distinct spin subensembles with absolute values of g‐factor spanning from 2.9 to 3.5 for electrons and from 0.5 to 1.6 for holes are resolved, revealing diverse localization environments. We measure the longitudinal spin relaxation times, $T_{1}$, reaching 2 ms and remaining in the μs range even for weakly localized carriers at cryogenic temperatures. The magnetic‐field dependence of $T_{1}$ is dominated by the random nuclear (Overhauser) fields with strengths of ∼0.5 mT for electrons and ∼5−12 mT for holes. The corresponding correlation times of the hyperfine field are determined by carrier hopping between shallow localization sites. The temperature dependence of $T_{1}$ reveals a weak localization potential of the charge carriers and shows a correlation between $T_{1}$ and the inhomogeneity of the spin ensemble. These results establish mixed‐A‐site perovskite single crystals as a promising solid‐state platform with long‐lived spin states for quantum information applications.

## Graphical abstract

Mixed‐cation ${\mathrm{MA}}_{x}$ ${\mathrm{FA}}_{1-x}$ ${\mathrm{PbI}}_{3}$ perovskite crystals reveal multiple discrete electron and hole spin subensembles associated with distinct localization environments. Optically detected magnetic resonance combined with resonant spin inertia uncovers carriers spin relaxation times reaching 2 ms and identifies nuclear‐field‐driven relaxation and carrier hopping as key spin relaxation mechanisms. These materials emerge as promising platforms for long‐lived solid‐state spin qubits.

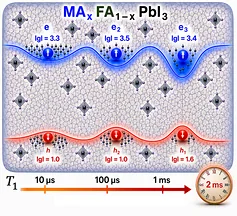

## 1 Introduction

Hybrid organic–inorganic lead halide perovskites (HOIPs) have rapidly emerged as one of the most intensively studied semiconductor classes in the past decade due to their exceptional optoelectronic properties [1, 2, 3, 4]. Beyond conventional photophysics, the possibility of optical spin orientation [5, 6] and the inverted band structure [7] with spins of electron and hole equal to 1/2, make them an ideal platform for exploring spin‐dependent phenomena. Compared with their extensively studied optical properties, spin‐related studies on HOIPs are still underdeveloped, especially for the mixed‐A‐site hybrid organic–inorganic perovskite crystals. One of the defining features of perovskite crystals is the presence of photo‐generated electrons and holes, spatially separated at different sites [8, 9, 10, 11], with distinct spin relaxation behavior and spin‐dependent parameters. Typically, two such spin signals corresponding to electrons and holes are observed in these perovskite crystals. However, a recent study of ${\mathrm{FAPbBr}}_{3}$ crystals surprisingly reported an additional electron spin species with slightly different g‐factor, localized in the potential fluctuations induced by crystal imperfections [12]. Therefore, the mechanism of carrier localization and its impact on the spin properties, especially spin relaxation, have remained an open question.

Spin relaxation is characterized by time $T_{1}$, reflecting the decay of the spin polarization along the magnetic field (diagonal element of the density matrix), and by spin coherence time $T_{2}$, reflecting the phase loss of the spin component rotating about the magnetic field (off‐diagonal element of the density matrix). A long longitudinal carrier spin relaxation time $T_{1}$ is critical for applications in quantum information technologies. Indeed, $T_{1}$ limits the spin coherence time $T_{2}$ [13, 14] and enables efficient dynamic nuclear polarization [8, 15]. Electron spin relaxation times reaching milliseconds and even seconds were reported only for strongly localized systems with suppressed spin‐orbit coupling, see the review in ref. [16]. In particular, $T_{1}$ approaching 1 ms was reported for inorganic perovskite nanocrystals (NCs) CsPb(Cl,Br)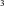 [17] and Ni^2+^‐doped CsPb(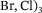 [18] at cryogenic temperatures. In bulk systems, the spin relaxation of charge carriers is usually enhanced by the spin‐orbit interaction, activating the Dyakonov‐Perel spin relaxation mechanism [19] and leading to spin relaxation times of a few nanoseconds. In perovskites, the Dyakonov–Perel spin relaxation mechanism is suppressed as a consequence of their unique property of spatial inversion symmetry [6, 20, 21]. In this respect, they are close to organic systems, where weak spin‐orbit interaction leads to spin lifetimes in the microsecond and even second range at 100–300 K [22, 23]. This allows one to expect long spin relaxation times of charge carriers even in bulk perovskite systems.

The so far reported $T_{1}$ values in bulk perovskite single crystals are ranging from tens to hundreds of nanoseconds, with examples including $T_{1}=37$ ns in ${\mathrm{MAPbI}}_{3}$ (MA = methylammonium) [24], $T_{1}=470$ ns in ${\mathrm{FAPbBr}}_{3}$ (FA = formamidinium) [12], and $T_{1}=53$ ns in ${\mathrm{CsPbBr}}_{3}$ [11]. These relatively short $T_{1}$ times are primarily related to the limited potential of pump‐probe and spin inertia [25] techniques for measuring μs‐long spin dynamics. Furthermore, these techniques cannot assign spin relaxation times $T_{1}$ to different spin species having different g‐factors, even to distinguish $T_{1}$ for electrons and holes. These disadvantages are resolved in the recently developed resonant spin inertia technique based on optically detected magnetic resonance (ODMR) with additional optical spin orientation of charge carriers [26, 27].

In this study, a comprehensive investigation of the spin relaxation dynamics in mixed‐A‐site hybrid ${\mathrm{MA}}_{x}{\mathrm{FA}}_{1-x}{\mathrm{PbI}}_{3}$ perovskite single crystals is conducted by taking advantage of the resonant spin inertia technique [26, 27]. We resolve multiple distinct electron and hole spin subensembles, each characterized by a unique g‐factor having absolute values in the range of 2.9−3.5 for electrons and 0.5−1.6 for holes. Such a wide, discrete g‐factor distribution directly reflects the coexistence of electrons and holes with different localization as well as nuclear environments. Remarkably, we observe a $T_{1}$ exceeding 2 ms in a hole subensemble at low temperature, nearly two to three orders of magnitude longer than previously reported in hybrid perovskites and in general in bulk semiconductors. Simultaneously, all other carrier spin subensembles show $T_{1}$ times of at least several microseconds. We also reveal resonances corresponding to quasi‐free electrons and holes. By analyzing their widths we extract the effective nuclear Overhauser fields of ∼0.4−0.8 mT acting on the electrons and ∼5−12 mT acting on the holes, consistent with the Pb‐dominated hyperfine interaction. Also, by analyzing the magnetic‐field dependence of $T_{1}$, which varies in the microsecond range, we determine the correlation times related the to nuclear fluctuations of about ∼0.04−0.4 μs for electrons and ∼1−15 μs for holes, respectively. These times correspond to hopping of carriers in a weak localizing potential. We show that an increase in the temperature from 1.6 to 7 K, leading to the carrier delocalization, results only in a moderate decrease in $T_{1}$ which remains in the microsecond range. These findings provide crucial insights into the spin dynamics in HOIP single crystals, and establish them as a promising platform for quantum technologies.

## 2 Results

### 2.1 Basic Spin Properties of ${\mathrm{MA}}_{x}{\mathrm{FA}}_{1-x}{\mathrm{PbI}}_{3}$ Perovskites: g Factors and $T_{1}$

The solution‐grown ${\mathrm{MA}}_{x}{\mathrm{FA}}_{1-x}{\mathrm{PbI}}_{3}$ perovskite crystals studied in this work were synthesized using the well‐established inverse crystallization method [28, 29]. We investigate two samples with MA content of x=0.4 and 0.8. All experiments are carried out at the temperature of 1.6 K unless specified otherwise. The reflectivity spectrum of the ${\mathrm{MA}}_{0.4}{\mathrm{FA}}_{0.6}{\mathrm{PbI}}_{3}$ crystal shown in Figure 1a has a maximum at 1.527 eV, which corresponds to the exciton‐polariton resonance. The photoluminescence (PL) spectrum has one line with the maximum at 1.524 eV, with a full width at half maximum of 7 meV, and a shoulder at 1.513 eV. The PL maximum has a slight Stokes shift of 3 meV with respect to the exciton‐polariton energy in the reflectivity spectrum.

**FIGURE 1:**
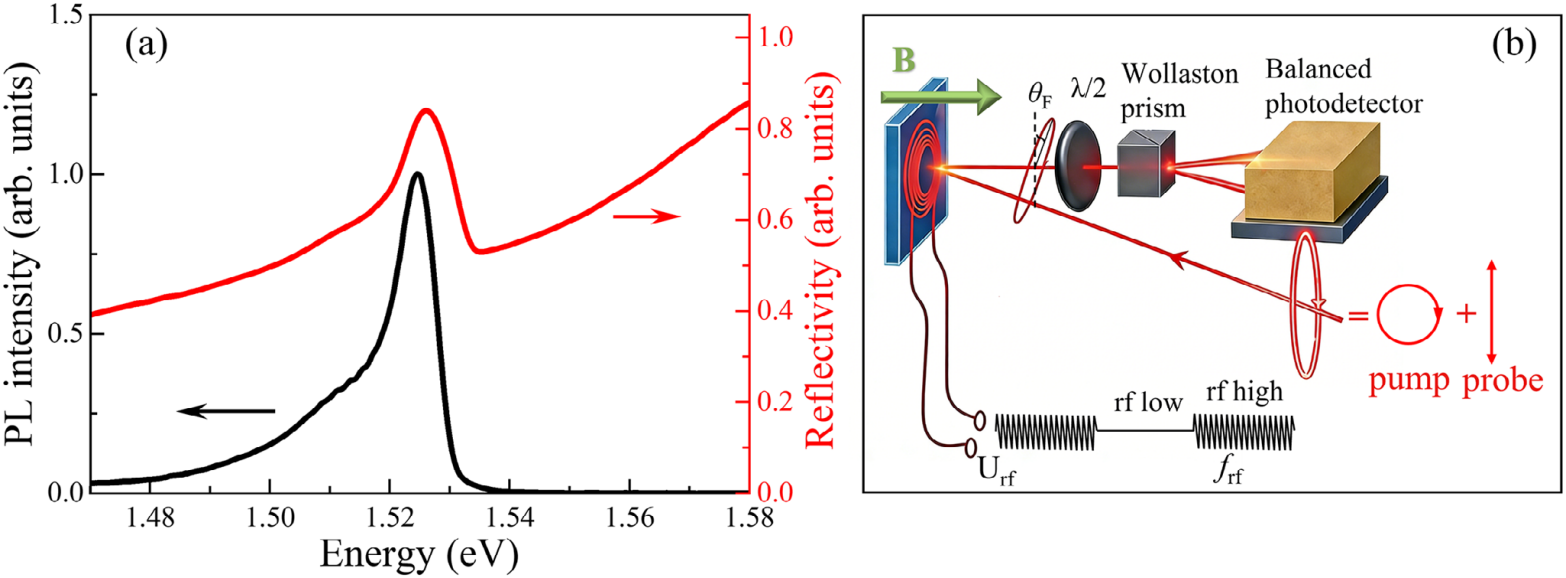
(a) Basic optical properties of the ${\mathrm{MA}}_{0.4}{\mathrm{FA}}_{0.6}{\mathrm{PbI}}_{3}$ crystal: photoluminescence spectrum (black) measured using continuous‐wave excitation with a photon energy of 3.06 eV and reflectivity spectrum (red). (b) Scheme of the experimental setup for measuring ODMR‐based resonant spin inertia.

To investigate the spin properties of charge carriers in the ${\mathrm{MA}}_{x}{\mathrm{FA}}_{1-x}{\mathrm{PbI}}_{3}$ crystals, the ODMR‐based resonant spin inertia technique is employed. Figure 1b shows the experimental setup. The technical details are given in the Experimental section. The carrier spin polarization generated by the circularly polarized component of the laser pulses accumulates along the external magnetic field B, which is applied in the Faraday geometry parallel to the sample normal (B∥k). The spin polarization is monitored via the Kerr rotation of the linearly polarized component. The accumulated spin polarization is destroyed by the rf field, when the spin precession Larmor frequency ($f_{L}$) matches the frequency of the rf field ($f_{\mathrm{rf}}$). Applying an rf field with a fixed frequency and scanning the external magnetic field, spin resonances can be recorded in Kerr rotation signals (ODMR signals), as shown in Figure 2a. ODMR spectra measured in this way show one narrow and one broad peak. Their resonance fields shift with changing the rf field frequency $f_{\text{rf}}$. The resonant frequency $f_{\text{rf}}=f_{L}$ as a function of the extracted magnetic fields corresponding to the maxima of the two peaks are plotted in Figure 2b. The dependencies are linear corresponding to

(1) $$hf_{L}=\left|g\right|\mu_{B}B\;,$$

where h is the Planck constant, and $\mu_{B}$ is the Bohr magneton. The slopes of the dependencies in Figure 2b give the two g‐factor values of 3.4 and 1.0. The k·p calculations and atomistic modeling [30, 31] suggest universal dependencies of the g factor on the bandgap for lead halide perovskites [32], which has been confirmed experimentally. According to this dependence, for the bandgap of 1.52 eV, $g_{e}>0$, $g_{h}<0$, and $|g_{e}\left|>\right|g_{h}|\;[$ 30, 31]. Therefore, we assign the larger $g_{e}=3.4$ to the electron, while attributing the smaller $g_{h}=-1.0$ to the hole, which is similar to the results obtained from time‐resolved optical orientation measurements, i.e. $g_{e}=3.27$ and $g_{h}=-1.02$ in ref. [21].

**FIGURE 2:**
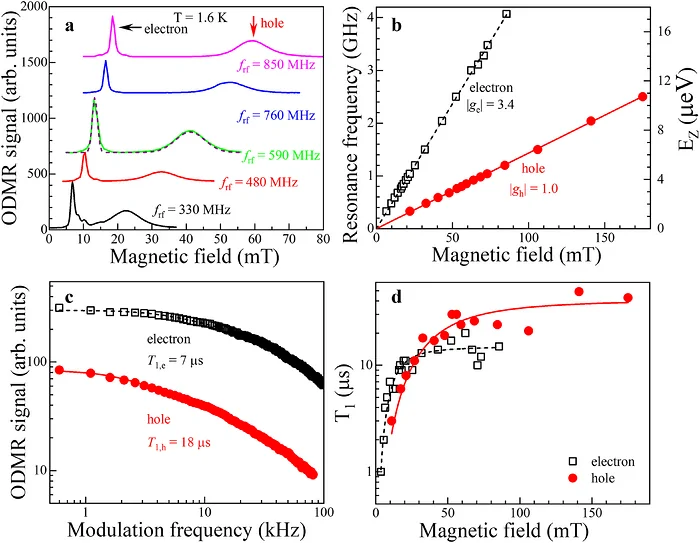
ODMR study of the ${\mathrm{MA}}_{0.4}{\mathrm{FA}}_{0.6}{\mathrm{PbI}}_{3}$ crystal. (a) ODMR spectra measured at different rf frequencies. The curves are vertically shifted for clarity. The dashed line shows the Gaussian fit. (b) Magnetic‐field dependence of the resonance frequencies corresponding to electron and hole in the ODMR spectra, with linear fits yielding $|g_{e}|=3.4$ and $|g_{h}|=1.0$. (c) ODMR signal as a function of the modulation frequency, $f_{\mathrm{mod}}$, at magnetic fields of 9.8 and 32.6 mT corresponding to the electron and hole resonances, respectively, at $f_{\mathrm{rf}}=480$ MHz. The lines show fits of the experimental data with Equations (S1)–(S4) in the Supporting Information. (d) Spin relaxation times, $T_{1}$, for electrons and holes as a function of magnetic field. Lines show fits of the experimental data with Equation (4). The laser photon energy is 1.528 eV, the laser power is 1 mW. T=1.6 K.

The width of the ODMR peak, ΔB, is determined by the spread of Larmor spin precession frequencies and provides information on the spin dephasing time $T_{2}^{*}$. The latter describes the dephasing of the Larmor precession in an inhomogeneous spin ensemble and typically is much shorter than the time $T_{2}$, which characterizes the coherence of a single spin. We assume a Gaussian distribution of the Larmor frequencies, which is justified by the Gaussian fit in Figure 2a, with ΔB being the standard deviation (σ). In this case the dephasing of the spin precession is described by a Gaussian decay $\propto \exp(-t^{2}/2T_{2}^{*2})$ [33] with the decay time given by the equation:

(2) $$T_{2}^{*}=\frac{\hbar }{\left|g\right|\mu_{B}\Delta B}.$$

The ODMR peak width ΔB and, correspondingly, $T_{2}^{*}$ are dominated by the random effective fields of the nuclear spin fluctuations and by the spread of the g factors, Δg [34]. The contribution of the g‐factor spread to the magnetic field linewidth increases linearly with magnetic field as $\Delta B_{g}=\left(\Delta g/g\right)B$. We do not observe a significant dependence of $T_{2}^{*}$ on the magnetic field over the measured range (see Figure S2 in the Supporting Information), suggesting a small contribution of the g‐factor spread. Thus, ΔB around 0.5 mT for electrons and around 4 mT for holes correspond to the random effective nuclear fields resulting from the hyperfine interaction. The corresponding spin dephasing times are $T_{2,e}^{*}=7$ ns for electrons and $T_{2,h}^{*}=2.8$ ns for holes. These values are in line with the results obtained for ${\mathrm{FA}}_{0.9}{\mathrm{Cs}}_{0.1}{\mathrm{PbI}}_{2.8}{\mathrm{Br}}_{0.2}$ crystals from pump‐probe Kerr rotation experiments [8]. We note that the hyperfine interaction for holes in perovskite crystals is stronger than that for electrons [8, 35], providing additional proof that the broader ODMR peak corresponds to holes, while the sharper peak corresponds to electrons.

For magnetic field strengths corresponding to the ODMR resonances, we can measure the longitudinal spin relaxation time $T_{1}$ of electrons and holes using the resonant spin inertia technique [26, 27]. To this end, we modulate the rf field at the frequency $f_{\mathrm{mod}}$ in the range from 0.1 to 100 kHz and measure ODMR signal as a function of $f_{\mathrm{mod}}$. Carrier spin polarization accumulates by optical pumping in the half of the period when the rf field is minimal, and then decays by the rf field action during the next half of the period (Figure 1b). The amplitude of the accumulated spin polarization is determined by the carrier spin lifetime $T_{1}$, when $1/f_{\mathrm{mod}}\gg T_{1}$, and by the time $1/f_{\mathrm{mod}}$, when $1/f_{\mathrm{mod}}\ll T_{1}$. Figure 2c shows the dependence of the ODMR signal (Kerr rotation amplitude) on the modulation frequency of the rf field, $f_{\mathrm{mod}}$. Increasing the modulation frequency beyond $1/T_{1}$ leads to a decrease in the ODMR signal for both electrons and holes allowing to estimate $T_{1}$. More quantitatively, $T_{1}$ can be determined using the spin inertia equation [17, 26, 27] for the spin polarization (Kerr rotation amplitude)

(3) $$S=\frac{AT_{1}^{2}}{\sqrt{1+{\left(2\pi T_{1}f_{\mathrm{mod}}\right)}^{2}}},$$

where the parameter A is the frequency‐independent coefficient determined by the excitation power and the amplitude of the rf field. We generalize this equation for a dynamics having two characteristic time scales (see Supporting Information) and use it for fitting the experimental dependencies in Figure 2c. The fits give spin relaxation times of $T_{1,e}=7$ μs and $T_{1,h}=18$ μs for electrons and holes, respectively. Note that an increase in the laser power leads to a reduction of $T_{1}$ through the additional perturbation of the spin system by the laser beam [17]. The dependencies of $1/T_{1}$ on the laser power P shown in Figure S3c for electrons and holes are linear and their extrapolation to P=0 yields $T_{1,e}=22$ μs and $T_{1,h}=88$ μs, respectively, for the undisturbed spin system. The measured $T_{1}$ for both electrons and holes are much longer than those of about 100 ns reported so far for other bulk perovskites [8, 10, 12].

It is interesting to examine the dependence of $T_{1}$ for electrons and holes spins on the magnetic field as shown in Figure 2d. Increase in the magnetic field strength leads to an increase in $T_{1}$. This is the typical behavior, as at low fields B the carrier spin dynamics is dominated by the Overhauser field of the nuclear spins varying with the characteristic time $\tau_{c}$, the correlation time of the fluctuating nuclear field. The increase in the external magnetic field B leads to suppression of the effect of the nuclear fluctuations and, therefore, $T_{1}$ increases. The analysis of the magnetic field dependence of $T_{1}$ allows us to evaluate the correlation time $\tau_{c}$ in our experiment. The described scenario was considered in ref. [36] leading to the following equation:

(4) $$T_{1}\left(B\right)=\frac{\tau_{s}}{1+{\left(\Delta_{N}/B\right)}^{2}\left(\tau_{s}/\tau_{c}\right)}.$$

Here $\Delta_{N}$ is the spread of the Overhauser nuclear field distribution $\pi^{-3/2}\Delta_{N}^{-3}\exp\left(-B_{N}^{2}/\Delta_{N}^{2}\right)$ and $\tau_{s}$ is the spin relaxation time in the absence of the nuclear fluctuations. This equation is valid for B exceeding $\Delta_{N}$ and the corresponding Larmor precession frequency exceeding $1/\tau_{c}$. Both conditions are fulfilled in our experiment. For the fit we assume values of the Overhauser field of 0.7 mT and 6 mT for electrons and holes, respectively, obtained from the widths of the ODMR peaks $\Delta_{N}=\sqrt{2}\Delta B$. From the reasonable fit shown in Figure 2d, we obtain the values of the effective nuclear correlation times of $\tau_{\text{c,e}}=0.04$ μs for the electrons and $\tau_{\text{c,h}}=0.9$ μs for the holes.

Next we investigate the spin dynamics of carriers at different temperatures ranging from 1.6 to 7.1 K, as shown in Figure 3. With increasing temperature, the ODMR signal shows a pronounced reduction in amplitude, see Figure 3a. $T_{1}$ decreases with increasing temperature for both electrons and holes, as shown in Figure 3b. Note that the $T_{1}$ for holes remains consistently larger than that for electrons across the investigated temperature range. Remarkably, even at T=7.1 K, the electron time $T_{1}$ remains as large as 2 μs. Note that for temperatures above 4 K a slightly higher laser photon energy is used to gain a better signal‐to‐noise ratio. This change in excitation energy results in a noticeable shift of the electron resonance position at 6.2 K. The temperature dependence of $T_{1}$ can be well fitted by an activation dependence [12]

(5) $$\frac{1}{T_{1}\left(T\right)}=\frac{1}{T_{1}\left(T=0\right)}+\gamma_{A}\exp\left(-\frac{E_{A}}{k_{B}T}\right)\;.$$

Here $\gamma_{A}$ is the thermal relaxation rate, $E_{A}$ is the activation energy, and $k_{B}$ is the Boltzmann constant. The corresponding fits to the experimental data shown by the lines in Figure 3b give values of the parameters $E_{\text{A,e}}=0.86$ meV for electrons and $E_{\text{A,h}}=0.91$ meV for holes. Such a small energy scale presumably corresponds to shallow potential fluctuations that localize the carriers. In addition, in Figure S4, we plot the temperature dependence of the spin dephasing times $T_{2}^{*}$ and fit them using the Equation (5). The fit yields distinctly different activation energies for electrons and holes: (*E*_A,e_ = 3.7 meV and *E*_A,h_ = 1.3 meV). The possible origin of these differences is discussed below in the Discussion section.

**FIGURE 3:**
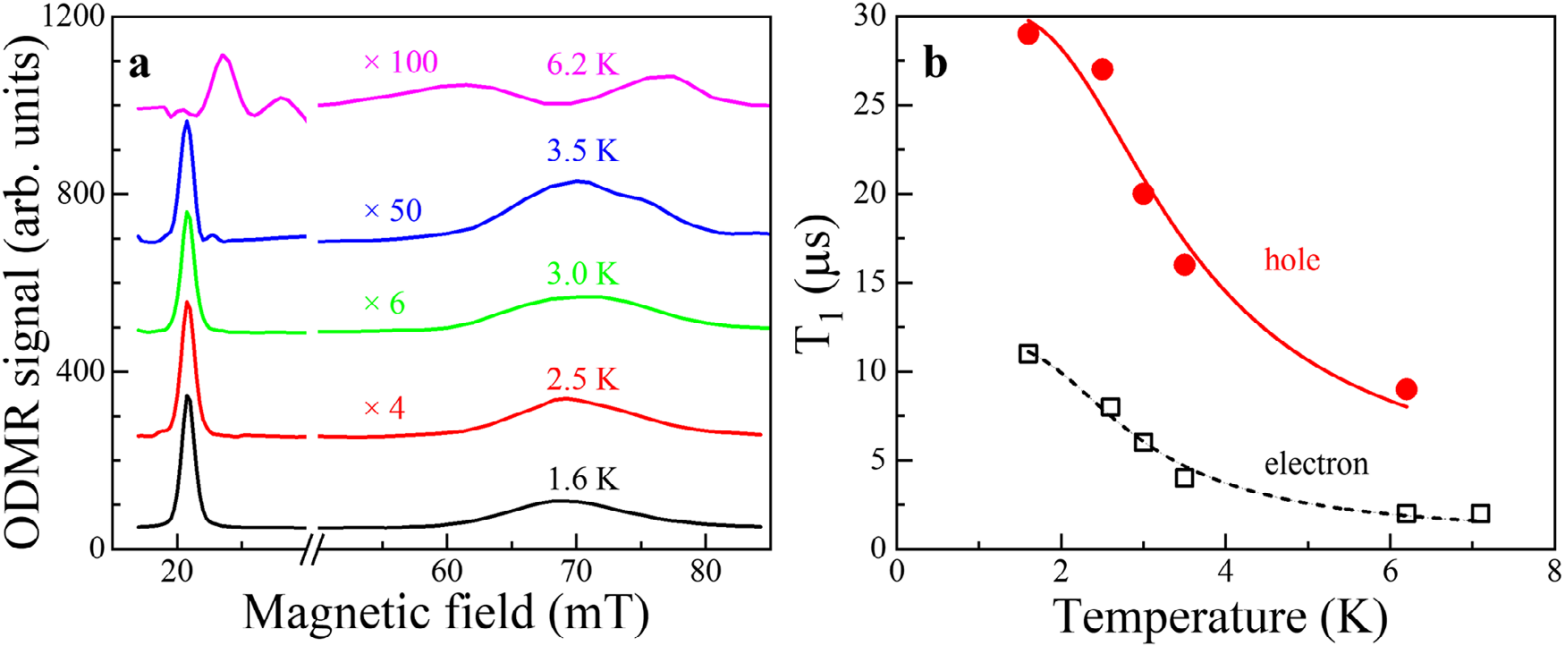
Temperature dependence for the MA_0.4_FA_0.6_PbI_3_ crystal. (a) ODMR spectra measured at different temperatures with the rf frequency fixed at 980 MHz. The curves are vertically shifted and multiplied by the indicated factors for clarity. (b) Temperature dependence of the longitudinal spin relaxation time $T_{1}$ for electron and hole resonances measured at magnetic fields of 20.6 and 68.5 mT, respectively, and $f_{\text{rf}}=980$ MHz. The lines are fits using Equation (5). For temperatures below 4 K, the laser photon energy is set to 1.528 eV, whereas for temperatures above 4 K, the laser photon energy is 1.530 eV. The laser power is 1 mW.

### 2.2 Multiple Distinct Spin States

A decrease in $f_{\mathrm{rf}}$ down to 150 MHz shifts the electron (labeled as e) and hole (labeled as h) resonances to smaller magnetic fields (Figure 4a). Surprisingly, this also leads to the appearance of new resonances in the ODMR spectrum (labeled as $e_{i}$ or $h_{i}$). The frequencies of the all observed resonances that increase linearly with magnetic field (Figure 4b), yield absolute g‐factor values of 3.1, 3.3, 3.6, 3.5, 1.0, 1.6, and 1.0. Note that the g‐factor values may slightly vary depending on the excitation energy and the excitation position on the crystal due to inhomogeneity. The four resonances with g‐factors around 3 likely correspond to distinct spin subensembles, arising from varying strengths of electron localization in potential fluctuations caused by crystal imperfections (e.g., local octahedral distortions), or binding to impurities or point defects [12]. Notably, for the additional resonance peaks ($e_{1}$, $e_{2}$ and $e_{3}$), the linear magnetic field dependencies of the resonance frequencies show offsets at zero field. These offsets are unlikely to originate from dynamical nuclear polarization [8], because they behave symmetrically upon reversing the magnetic field direction from negative to positive, as demonstrated in Figure S5b.

**FIGURE 4:**
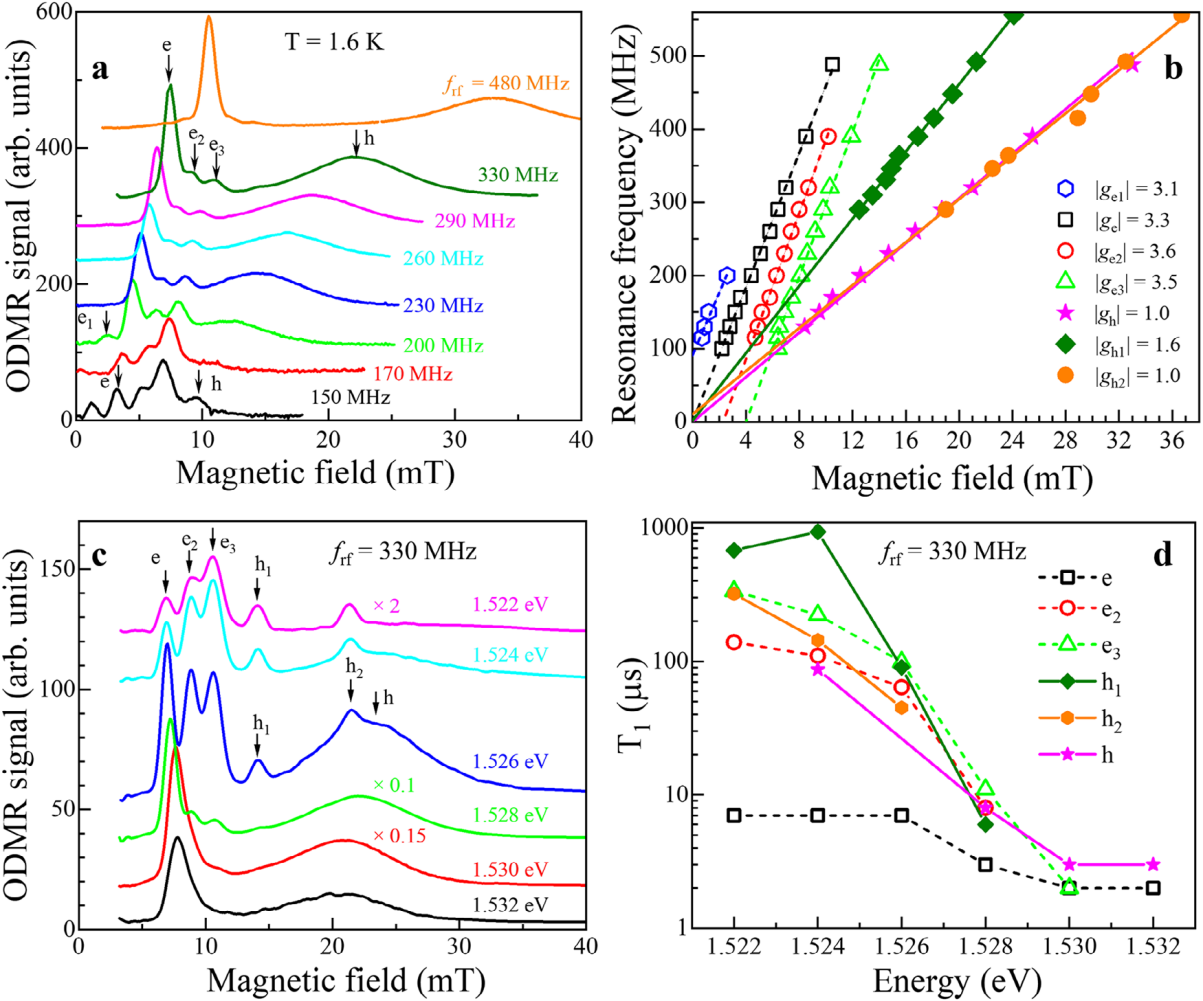
Multiple distinct spin states in the ${\mathrm{MA}}_{0.4}{\mathrm{FA}}_{0.6}{\mathrm{PbI}}_{3}$ crystal. (a) ODMR spectra measured at different rf frequencies. The curves are vertically shifted for clarity. (b) Magnetic field dependence of the resonance frequencies of the ODMR peaks observed in the panel a with linear fits shown by the lines. The laser photon energy is 1.528 eV. The data for $h_{1}$ and $h_{2}$ are extracted from the data presented in Figure S5 with the excitation laser energy of 1.524 eV. (c) ODMR spectra at different laser photon energies at fixed *f*_rf_ = 330 MHz. (d) Spin relaxation times, $T_{1}$, for different electron and hole resonances as a function of the excitation energy at fixed *f*_rf_ = 330 MHz. The laser power is 2 mW, T=1.6 K.

Interestingly, the contribution of different resonances to the ODMR spectrum depends not only on the rf frequency, but also on the optical transition energy. In the following, we investigate the evolution of the ODMR spectrum at a fixed rf frequency of *f*_rf_ = 330 MHz as a function of the laser photon energy, shown in Figure 4c. Note that the electron spin subensemble with the g‐factor of 3.1 (marked as $e_{1}$ in Figure 4b) is not detectable at this rf frequency. At the highest photon energy, the spectrum exhibits only a narrow electron peak (e) and a broad hole peak (h) that were discussed above in the manuscript. A decrease in photon energy leads to the suppression of these peaks and the appearance of other narrow, satellite, peaks. The ODMR signal exhibits a resonant enhancement at the excitation energy of 1.528 eV. Decrease in the laser photon energy to below 1.528 eV leads to the appearance of a new resonant peak ($h_{1}$) at 14.7 mT, corresponding to $|g_{\text{h1}}|=1.6$, so that it may originate from a hole spin subensemble. Also the broad hole peak becomes accompanied by a more narrow peak ($h_{2}$) at the field of 21.5 mT with $|g_{\text{h2}}|=1.0$, which presumably also has hole origin. Remarkably, the spin dephasing time $T_{2}^{*}$ estimated from the ODMR peak width reaches 17 ns for the $h_{2}$ spin subensemble at the excitation energy of 1.522 eV (see Figure S6), which exceeds previously reported hole spin dephasing times for perovskite materials. Interestingly, the electron peak e slightly shifts toward lower magnetic fields with decreasing laser photon energy in contrast to the hole peak h, which shifts toward higher fields.

Next, we determine the $T_{1}$ related to the different ODMR resonances as function of the laser energy (Figure 4d). As the laser energy decreases from 1.532 to 1.522 eV, the time $T_{1}$ for all spin subensembles significantly increases. Specifically, the spin relaxation time of the electron resonance denoted as e with $|g_{e}|=3.3$ increases only by a factor of 4, from 1.7 μs to 7 μs. In contrast, the electron spin subensembles with $|g_{\text{e2}}|=3.6$ (denoted $e_{2}$) and $|g_{\text{e3}}|=3.5$ (denoted $e_{3}$) exhibit a significantly larger increase in $T_{1}$, approximately ten‐fold and thirty‐fold, respectively. For all resonances except of $e_{1}$, the time $T_{1}$ can reach several hundreds of μs (and even 1 ms for $h_{1}$) at the smallest excitation energy of 1.522 eV. At the laser energy of 1.524 eV, the $h_{2}$ spin subensemble, corresponding to a narrow ODMR resonance, exhibits a $T_{1}$ nearly 2 times longer than that for the h spin subensemble showing up as broad peak. The times $T_{1}$ for the additional resonances also show magnetic field dependence which is presented in Figure S7.

Similar experimental appearances are found for the ${\mathrm{MA}}_{0.8}{\mathrm{FA}}_{0.2}{\mathrm{PbI}}_{3}$ crystal having the MA composition of 0.8 (compared to 0.4), which is discussed in the Supporting Information. These results are shown in Figure S8.

## 3 Discussion

Using the ODMR‐based technique we have observed a number of spin resonances for the ${\mathrm{MA}}_{x}{\mathrm{FA}}_{1-x}{\mathrm{PbI}}_{3}$ crystals with x=0.4 and 0.8 and measured the basic spin parameters related to these resonances: g factor, spin precession frequency offset at B=0, longitudinal spin relaxation time $T_{1}$, and inhomogeneous spin dephasing time $T_{2}^{*}$. These spin parameters are summarized in Tables 1 and 2 for x=0.4 and 0.8, respectively. Here the uncertainty of g factors and frequency offsets takes into account the spread of these parameters for measurements in different points of the sample and at different laser energies.

**TABLE 1:** Measured and evaluated parameters of the studied perovskite crystals ${\mathrm{MA}}_{0.4}{\mathrm{FA}}_{0.6}{\mathrm{PbI}}_{3}$ at T=1.6 K. The values of $T_{1}$ and ΔB are extracted at the laser photon energy of 1.524 eV, with the rf frequency of 330 MHz. Note that the ΔB of the $e_{1}$ subensemble is extracted from the data shown in Figure 4a using the rf frequency of 200 MHz.

State	|g|	Offset (MHz)	$T_{1}$ (μs)	ΔB (mT)	$T_{2}^{*}$ (ns)	$\tau_{c}$ (μs)
e	3.3±0.1	0±10	7	0.5	6.9	0.04
$e_{1}$	3.1±0.3	90±10	—	0.5	7.3	—
$e_{2}$	3.5±0.2	−110±20	110	0.5	6.5	—
$e_{3}$	3.4±0.1	−180±20	220	0.7	4.5	—
h	1.02±0.05	0±20	90	4.8	2.1	0.9
$h_{1}$	1.61±0.04	10±10	940	0.6	12	—
$h_{2}$	1.03±0.07	10±30	140	0.7	15	—

**TABLE 2:** Measured and evaluated parameters of the studied perovskite crystal ${\mathrm{MA}}_{0.8}{\mathrm{FA}}_{0.2}{\mathrm{PbI}}_{3}$ at T=1.6 K. The $T_{1}$ and ΔB are extracted at the laser photon energy of 1.617 eV, with the rf frequency of 330 MHz.

State	|g|	Offset (MHz)	$T_{1}$ (μs)	ΔB (mT)	$T_{2}^{*}$ (ns)	$\tau_{c}$ (μs)
e	2.9±0.1	10±10	6	0.6	6.5	0.4
$e_{2}$	3.4±0.2	−160±40	150	0.7	4.8	—
$e_{3}$	3.1±0.1	−190±20	230	1.1	3.3	—
h	0.50±0.05	10±10	160	11.7	1.9	15
$h_{1}$	1.2±0.1	50±30	2100	1.1	8.6	—
$h_{2}$	0.9±0.1	10±10	1140	1.0	13	—

Across a wide range of magnetic fields (rf frequencies) and laser energies we observe resonances, denoted in the tables as e and h with absolute g factor values close to 3 and 1, which we attribute to electrons and holes, respectively. They have drastically different widths of about 1 and 10 mT for electrons and holes, respectively, which correspond to the spread of the Overhauser fields of the nuclear spin fluctuations. The hyperfine interaction is much stronger for holes rather than for electrons in perovskites [8, 15, 37], which is in line with our experimental results.

We have measured long spin relaxation times $T_{1}$ reaching tens of μs for both electrons and holes, which is surprising for bulk semiconductors. We note that, in general, in our experiments we have measured spin lifetimes that are contributed by both the carrier lifetime and the actual spin relaxation time $T_{1}$. However, the strong dependence of the measured time on the magnetic field suggests that it is determined mostly by $T_{1}$ and, thus, we deal with resident carriers having a very long lifetime. The same is true for the additional spin resonances, which also have a strong dependence of the spin lifetime on the magnetic field (see Figure S7). For the resident carriers the mechanism of optical orientation and spin detection via Faraday rotation is essentially the same as was described in the work [38] for singly charged quantum dots. The times $T_{1}$ increase with magnetic field (Figure 2d and Figure S8c) due to the suppression of the time‐varying nuclear spin fluctuations. These dependencies allow us to estimate the nuclear field correlation times $\tau_{c}$ of 0.04 μs (0.4 μs) for electrons and 0.9 μs (15 μs) for holes for the samples with x=0.4 (x=0.8). For strongly localized carriers, $\tau_{c}$ is determined by the nuclear spin dynamics. However, in lead halide perovskites, the electrons and holes interact with different nuclear species, with the holes primarily coupled to the Pb nuclei and the electrons coupled to both the Pb and I nuclei [8]. Since the nuclear spin dynamics of Pb and I can differ, a difference in $\tau_{c}$ between electrons and holes can be expected even in the strongly localized regime. For weakly localized carriers $\tau_{c}$ may be determined by the carrier hopping between potential traps having different nuclear environments, if the time for these hoppings is shorter than the evolution time of the nuclear polarization. In our case, we have strongly different $\tau_{c}$ for electrons and holes. Also, $\tau_{c}$ is rather different for samples with different MA concentration x. These facts suggest that $\tau_{c}$ is determined mostly by carrier hopping rather than by nuclear spin dynamics, which is expected to be weakly dependent on carrier type and x. This gives us evidence that we deal with carriers weakly localized in shallow potentials, and that the carriers are more delocalized in the sample with reduced MA content. Thus, at low magnetic fields, spin relaxation is dominated by the hyperfine interaction with nuclear spins. At high magnetic fields, spin relaxation might be contributed by the mechanisms related to spin‐orbit interaction. As mentioned earlier, inversion symmetry of the perovskite lattice suppresses spin‐orbit coupling and, thus, the Dynakonov–Perel spin relaxation mechanism, which is usually very efficient for free carriers [6, 20, 21]. However, lattice imperfections, residual electric fields, and defects may break this symmetry, especially for localized carriers, which may result in spin‐orbit coupling and the corresponding relaxation mechanisms, both of the Dyakonov–Perel type, where spin relaxes as a result of carrier motion and frequent scattering slows down the relaxation, and the Elliott–Yafet type, where spin relaxes as a result of scattering [39]. Such relaxation may take place for carriers hopping between different localization sites, just as in our case. An additional spin relaxation mechanism, especially important at elevated temperatures, is related to the two‐phonon Raman process as described in Ref. [40].

Another confirmation of a weak localization by shallow potentials comes from the temperature dependence of $T_{1}$ (Figure 3b), which shows an activation behavior with rather small energies of about 1 meV for both electrons and holes. This energy can be related to the depth of carrier localization potential. We also highlight the decrease in the ODMR signal amplitude when the temperature is increased (Figure 3a). Note that the accumulated spin polarization is proportional to $T_{1}^{2}$ [Equation (3)] [26, 27]. However, the decrease in $T_{1}$ cannot fully account for the decrease in the ODMR signal. For example, when the temperature is increased from 1.6 to 3.5 K, the electron $T_{\text{1,e}}$ decreases from 11 to 6 μs, corresponding to a three‐fold decrease in $T_{1}^{2}$, while the experiment reveals a 50‐fold decrease in the ODMR signal. This dominant signal suppression can be related to electron and hole delocalization with their subsequent recombination, which, thus, reduces the number of resident carriers that can be oriented optically.

The other observation following from the temperature dependence of the ODMR spectra is the broadening of the ODMR resonances with temperature (Figure 3a), quantified as a decrease in the inhomogeneous dephasing time $T_{2}^{*}$ (Figure S4). In a conventional scenario, $T_{2}^{*}$ is expected to remain nearly temperature independent as long as $T_{1}\gg T_{2}^{*}$, and to decrease only at elevated temperatures when $T_{1}$ approaches the nanosecond‐long $T_{2}^{*}$ [34]. In contrast, in our measurements $T_{2}^{*}$ shows a pronounced temperature dependence, even though $T_{1}\gg T_{2}^{*}$. One possible explanation is the temperature dependence of the inhomogeneity in the system. This inhomogeneity may result in a broad distribution of $T_{1}$ in the spin ensemble. At low temperature, the measured signal is dominated by the carriers with the longest $T_{1}$ [see Equation (3)], which could correspond to a narrow distribution of precession frequencies and thus a longer $T_{2}^{*}$. Increase in the temperature primarily leads to the suppression of the longest $T_{1}$ and extension of the spin ensemble that dominates the ODMR signal. The larger ensemble shows a larger spread of the Larmor frequencies, resulting in shorter $T_{2}^{*}$. This can also explain the different activation energies for $T_{1}$ and $T_{2}^{*}$.

In both samples we also find multiple spin resonances which accompany the main electron and hole peaks. These resonances show up under specific conditions, namely at low fields and Larmor precession frequencies (Figure 4a) and at laser energies below the exciton resonance (Figure 4c). Resonances that have g factors close to that of electron (hole) are attributed to electron (hole) spin subensembles and labeled as $e_{i}$ ($h_{i}$). Electron satellite resonances show an offset in the dependence of their frequency on magnetic field (Figure 4b, Figure S8b, Tables 1 and 2). One can attribute these offsets to internal fields, locally experienced by the corresponding carrier spin subensembles. In particular, the offset may be related to the effective field arising from the exchange interaction in an exciton [17] or to the Overhauser field of the nuclear spin fluctuations [41, 42]. However, these cases would be characterized by a positive offset, i.e., a finite frequency at B=0 which is the case only for the $e_{1}$ resonance in Figure 4b. One should mention also the dynamic nuclear polarization, where optical pumping with certain helicity results in the Overhauser field directed along the external field, but independent of its sign. An internal field $B_{i}$, if it is independent of the sign of the external field (B vs −B), should effectively shift the magnetic field dependence horizontally. However, this interpretation is contradictory to the experimental observations: the corresponding dependence is symmetric when the magnetic field is scanned from negative to positive values and is shifted vertically to lower frequencies (Figure S5b). This may take place if the internal magnetic field changes sign with the external magnetic field.

We emphasize the ultralong spin relaxation time of these satellite resonances, which becomes further enhanced for decreasing energy of the corresponding optical transition (Figure 4c). The longest $T_{1}$ reaches 2.1 ms for the $h_{1}$ transition in the ${\mathrm{MA}}_{0.8}{\mathrm{FA}}_{0.2}{\mathrm{PbI}}_{3}$ sample (see Figure S8d and Table 2). The fact that these resonances are observed at decreasing laser energy suggests their localization character. On the other hand, for strongly localized carriers the effect of random nuclear spins is expected to be enhanced. This should lead to a decreasing $T_{1}$, especially at low fields (Figure 2d) and to broadened ODMR resonances. In fact, all new resonances, although appearing at low B, are characterized by an exceptionally long $T_{1}$ and a narrow ODMR spectrum, even those having hole character (Figure 4c and Figure S8a). We note that in the extreme limit of strong localization, a carrier may strongly interact with only a few nuclei. This interaction, instead of causing spin‐resonance broadening, can lead to the formation of several discrete (and narrow) spin energy levels in the interacting carrier‐nuclei system. This scenario is observed for strongly localized spin states, e.g., in rare earth ions [43, 44].

We note that in the ${\mathrm{FAPbI}}_{3}$ the electron and hole g factors at the bandgap energy of 1.48 eV are $g_{e}=3.45$ and $g_{h}=-1.13$ [45]. In ${\mathrm{MAPbI}}_{3}$ the electron and hole g factors are anisotropic and span the range $g_{e}=2.5÷3.0$, $g_{h}=-\left(0.3÷0.7\right)$ at the bandgap energy of 1.637 eV [24, 46]. It is instructive to compare these g factors to those presented in Tables 1 and 2 for the ${\mathrm{MA}}_{x}{\mathrm{FA}}_{1-x}{\mathrm{PbI}}_{3}$ compounds. For the quasi‐free electron state e $|g_{e}|=3.3$ for ${\mathrm{MA}}_{0.4}{\mathrm{FA}}_{0.6}{\mathrm{PbI}}_{3}$, which is closer to the value in ${\mathrm{FAPbI}}_{3}$ and $|g_{e}|=2.9$ for ${\mathrm{MA}}_{0.8}{\mathrm{FA}}_{0.2}{\mathrm{PbI}}_{3}$ which is closer to the value in ${\mathrm{MAPbI}}_{3}$. The same tendency holds for the hole h state, for which $|g_{h}|=1.0$ for ${\mathrm{MA}}_{0.4}{\mathrm{FA}}_{0.6}{\mathrm{PbI}}_{3}$ and $|g_{h}|=0.5$ for ${\mathrm{MA}}_{0.8}{\mathrm{FA}}_{0.2}{\mathrm{PbI}}_{3}$. The values of |g| as a function of MA content are summarized in Figure 5. The g factors for satellite electron resonances $e_{1}$, $e_{2}$, $e_{3}$ are close to that of e and within measurement error fall within the range from 2.5 to 3.45 corresponding to an MA content from 1 to 0. For the hole‐like satellites $h_{2}$ has g factor within the expected g factor range of MA content from 1 to 0, while $h_{1}$ has a |g| value larger than expected. The satellite states can be related to the crystal defects having fluctuations of MA content, different type or orientation of the crystal lattice. We note that carrier localization in such sites can lead to significant deviations of the g factor from the value predicted by the bandgap energy [47, 48].

**FIGURE 5:**
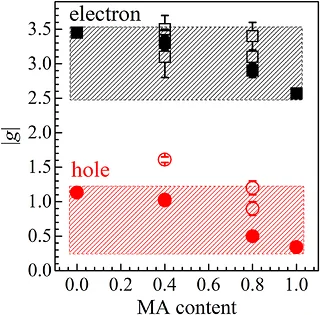
Absolute value of g factors for electrons (black squares) and holes (red circles) as a function of the MA content in ${\mathrm{MA}}_{x}{\mathrm{FA}}_{1-x}{\mathrm{PbI}}_{3}$ crystals. For ${\mathrm{FAPbI}}_{3}$ crystal the data is taken from the work [45]. For ${\mathrm{MAPbI}}_{3}$ crystal with anisotropic g factor we take the lowest |g| values from the work [24]. Open symbols show |g| for the satellite resonances. Dashed regions show ranges of g factors for MA content spanning from 0 to 1.

In fact, the relative number of these satellite states is quite small, and they are revealed in the resonance spin inertia technique due to their long $T_{1}$ time. Indeed, the signal in our experiment is proportional to $T_{1}^{2}$ [Equation (3)] [26, 27]. Considering that the ratio of the $T_{1}$ times between the satellite and the main resonances is about 10−100, we can conclude that the corresponding ratio of spin populations is about $10^{-4}-10^{-2}$. This complicates the observation of these satellite resonances by other techniques, e.g., time‐resolved Faraday/Kerr rotation.

## 4 Conclusions

In summary, we have conducted a comprehensive ODMR investigation of the spin properties in mixed‐A‐site hybrid organic–inorganic perovskite ${\mathrm{MA}}_{x}{\mathrm{FA}}_{1-x}{\mathrm{PbI}}_{3}$ single crystals with *x* = 0.4 and 0.8. Across a wide range of magnetic fields and optical transition energies, we have observed electron and hole resonances with g factor absolute values of 3.3 (2.9) and 1.0 (0.5) for electrons and holes, respectively, in the sample with x=0.4 (0.8). The widths of the ODMR peaks allow one to evaluate Overhauser fields of approximately 0.5−0.8 mT for electrons and 4−12 mT for holes. These resonances have longitudinal spin relaxation times $T_{1}$ reaching hundreds of microseconds, which confirms the absence of the Dyakonov–Perel spin relaxation mechanism due to the lattice symmetry in this system. By analyzing the magnetic‐field dependence of $T_{1}$, we have evaluated nuclear field correlation times $\tau_{c}$, which are about 0.04 μs for electrons and 0.9 μs for holes in ${\mathrm{MA}}_{0.4}{\mathrm{FA}}_{0.6}{\mathrm{PbI}}_{3}$ crystal and about order of magnitude longer in ${\mathrm{MA}}_{0.8}{\mathrm{FA}}_{0.2}{\mathrm{PbI}}_{3}$ crystal. These times are dominated by carrier hopping in a weak localizing potential landscape and indicate that carriers are more delocalized in the sample with reduced MA content. The carriers are also delocalized by increasing the temperature from 1.6 to 7 K, which leads to a moderate decrease in $T_{1}$ and pronounced decrease in the resident carriers density.

At low rf field frequencies (and magnetic fields), we have resolved a set of carrier spin subensembles, each with a distinct g‐factor absolute values spanning the range of 2.9−3.5 for electrons and of 0.5−1.6 for holes. The relative amplitude of the different peaks strongly depends on the optical transition energy, suggesting their origin from different subensembles of electrons and holes with different degrees of localization and distinct hyperfine environments. Furthermore, all detected carrier subensembles exhibit micro‐to‐millisecond $T_{1}$ times, with a record value of 2.1 ms, underscoring the exceptionally slow spin relaxation in mixed‐cation hybrid perovskite single crystals. These findings establish hybrid organic–inorganic perovskite single crystals as a compelling solid‐state platform for spin physics, revealing a complex interplay of *g*‐factor dispersion, carrier localization, and hyperfine interaction, and combining long spin lifetimes with optical control in a technologically relevant material system.

## 5 Experimental Section

### 5.1 Samples

The ${\mathrm{MA}}_{0.4}{\mathrm{FA}}_{0.6}{\mathrm{PbI}}_{3}$ and ${\mathrm{MA}}_{0.8}{\mathrm{FA}}_{0.2}{\mathrm{PbI}}_{3}$ single crystals were synthesized according to the well‐established inverse crystallization method [28, 29]. This method reliably yields high‐quality single crystals with well‐defined facets and long carrier diffusion lengths [49]. X‐ray diffraction measurements further confirm the excellent structural quality of these perovskite single crystals [50]. The studied ${\mathrm{MA}}_{x}{\mathrm{FA}}_{1-x}{\mathrm{PbI}}_{3}$ single crystals with x=0.4 and 0.8 were synthesized from appropriately mixed MAI, FAI, and ${\mathrm{PbI}}_{2}$ perovskite precursors. The precursors were injected between two polytetrafluoroethylene coated glasses and slowly heated to 120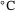. The samples have square shapes that reach about 2×2 mm in the (001) crystallographic plane and a thickness of about 30 μm.

### 5.2 Optical measurements

The photoluminescence of the crystals is dispersed by a 0.5 m monochromator and detected with a charge‐coupled‐device (CCD) camera following its excitation with a 3.06 eV continuous‐wave (cw) diode laser. Reflectivity spectra were measured using a halogen lamp in back‐reflection geometry.

### 5.3 ODMR measurements

The experimental scheme used to measure the spin resonances and the longitudinal spin relaxation time $T_{1}$ is shown in Figure 1b [26, 27]. We use a Coherent Chameleon Discovery laser system emitting 100 fs pulses at a repetition rate of 80 MHz, which wavelength is tunable over a wide spectral range. To reduce the spectral width to ∼1 nm, a grating‐based pulse shaper is used. The sample is placed in a He‐bath cryostat with superconducting coils. At T<4.2 K the sample was immersed in a liquid helium, while at higher temperatures it was held in a helium gas. The magnetic field *B* is applied parallel to the sample normal (B∥k) (Faraday geometry). Optical spin orientation and spin polarization probing are performed using the same laser beam with elliptical polarization. The circular polarization component of the beam serves as pump for the carrier spins, while the linear component is used to probe the spin polarization via the Kerr rotation effect [14, 17, 27]. The Kerr rotation is detected using a Wollaston prism, splitting the beam into two orthogonally polarized beams of approximately equal intensity which are detected by a balanced photodetector. The rf magnetic field is applied using a small coil near the sample surface. The current through the coil is driven by a function generator, which creates a sinusoidal voltage with frequency $f_{\mathrm{rf}}$, ranging from 100 to 4500 MHz. The generator output is modulated sinusoidally at frequency $f_{\mathrm{mod}}$, ranging from 0.1 to 100 kHz for synchronous detection with a lock‐in amplifier. Thus, we measure the ODMR signal as a difference between Kerr rotation amplitude with the rf field at low and high level, which in turn is proportional to the corresponding difference ΔS in the spin polarization. The spin polarization created by resonant laser excitation accumulates along the external magnetic field B. The spins are addressed by the rf field only if their Larmor precession frequency $f_{L}$ matches $f_{\mathrm{rf}}$. To implement the resonant spin inertia method, the rf field modulation frequency $f_{\mathrm{mod}}$ is varied. As $f_{\mathrm{mod}}$ increases, the corresponding modulation period becomes shorter than $T_{1}$, leading to a measurable reduction in the spin modulation amplitude. This dependence allows us to evaluate the $T_{1}$ time by means of Equation (3) [26].

## Conflicts of Interest

The authors declare no conflicts of interest.

## Supporting information


**Supporting File**: advs76770‐sup‐0001‐SuppMat.pdf.

## Data Availability

The data that support the findings of this study are available from the corresponding author upon reasonable request.
