# Supplementary material for: Millisecond Spin Relaxation Times of Distinct Electron and Hole Subensembles in MA x FA1−x PbI3 Perovskite Crystals
Source: Adv Sci (Weinh). 2026 Jul 28;13(58):e76770. doi: 10.1002/advs.76770 (PMC13410821; doi:10.1002/advs.76770)
Supplement: Supplementary file 1 [file ADVS-13-e76770-s001.pdf]

Supporting Information: Millisecond spin relaxation times of distinct electron and hole subensembles in $MA_xFA_{1-x}PbI_3$ perovskite crystals

Rongrong Hu*[1,2], Sergey R. Meliakov[1], Dmitri R. Yakovlev[1], Bekir Turedi[3,4], Maksym V. Kovalenko[3,4], Manfred Bayer[1,5], and Vasilii V. Belykh*[1]

[1]Experimentelle Physik 2, Technische Universität Dortmund, 44227 Dortmund, Germany
[2]School of Science, Shanghai Institute of Technology, 201418 Shanghai, China
[3]Laboratory of Inorganic Chemistry, Department of Chemistry and Applied Biosciences, ETH Zürich, CH-8093 Zürich, Switzerland
[4]Laboratory for Thin Films and Photovoltaics, Empa-Swiss Federal Laboratories for Materials Science and Technology, CH-8600 Dübendorf, Switzerland
[5]Research Center FEMS, Technische Universität Dortmund, 44227 Dortmund, Germany

*Email: rongrong.hu@tu-dortmund.de, vasilii.belykh@tu-dortmund.de

S1. Generalization of the spin inertia equation for two carriers subensembles

For the case of two electron or hole subensembles having similar g-factors, but different spin relaxation times $T_{1,1}$ and $T_{1,2}$, the equation describing the resonant spin inertia derived in Ref. [1] can be generalized. We detect the spin signal in the X and Y channels of the lock in amplifier in phase with the rf field modulation and with a $\pi/2$ phase shift, respectively. These signals should add linearly for the two subensembles:

$$X = \sum_{i=1}^{2} \frac{A_i T_{1i}^2}{1 + 4\pi^2 T_{1i}^2 f_{\mathrm{m}}^2} \tag{S1}$$

$$Y = \sum_{i=1}^{2} \frac{2\pi T_{1i}^2 f_m^2 A_i}{1 + 4\pi^2 T_{1i}^2 f_{\mathrm{m}}^2} \tag{S2}$$

The total signal depicted in Figure 2c is calculated as

$$S = \sqrt{X^2 + Y^2}. \tag{S3}$$

Then, the average longitudinal spin relaxation time T_1 can be calculated via the intensity-weighted method, using the following equation

$$T_{1,avg} = \frac{\sum_{i=1}^{2} A_i T_{1i}^2}{\sum_{i=1}^{2} A_i T_{1i}}. \tag{S4}$$

To visualize the contributions of the individual components, we plot the two components separately together with the resulting spin inertia curve in Figure S1.

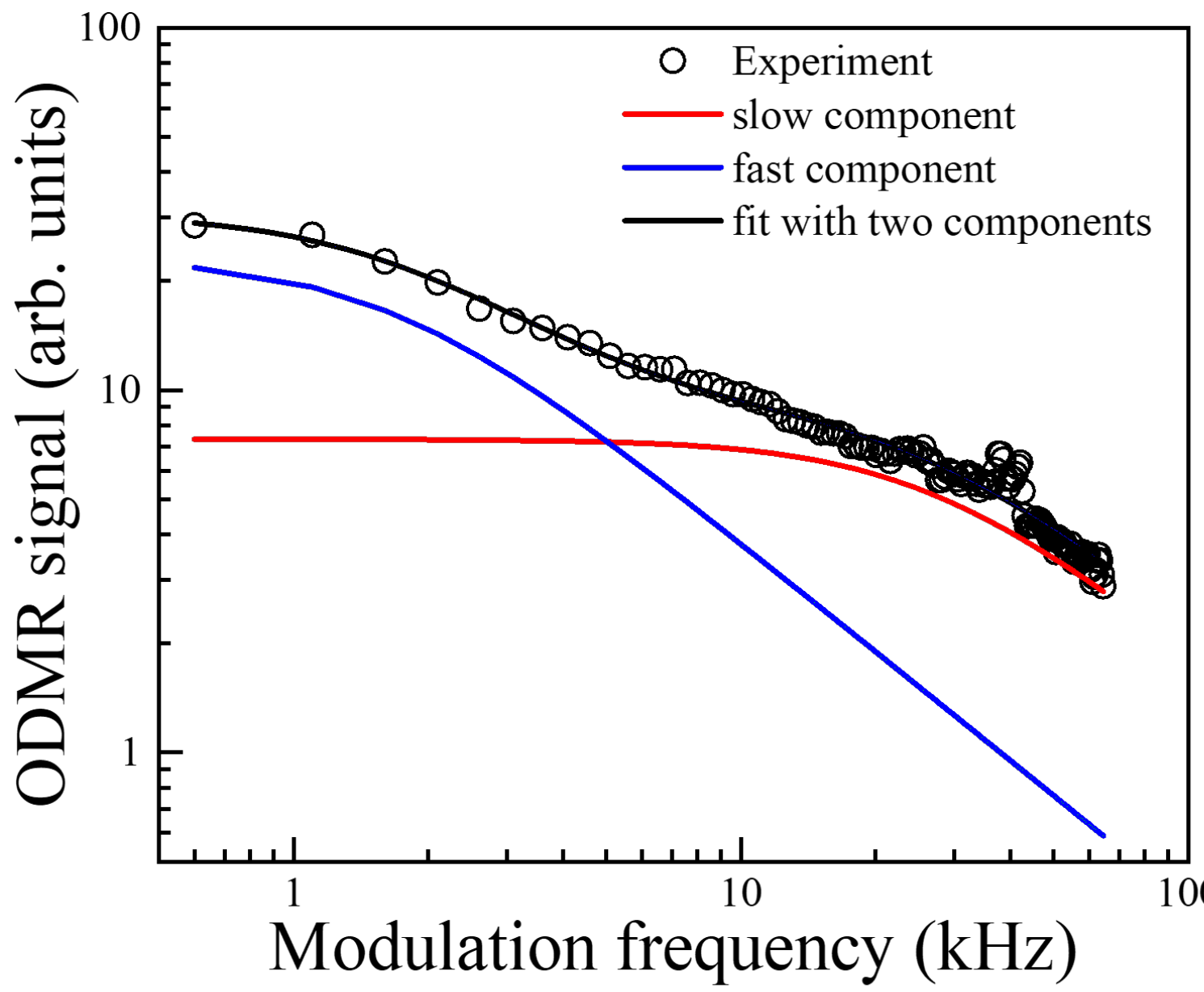

Figure S1: ODMR signal as function of the modulation frequency, f_{mod}, for holes in the $\mathrm{MA_{0.4}FA_{0.6}PbI_3}$ crystal at $B = 106$ mT. The black line is a fit with two components using the equations (S1-S3). The blue and red lines show the individual contributions of the slow and fast components, respectively. $T = 1.6$ K.

S2. Magnetic field dependencies of the spin dephasing time and the width of the ODMR peak in the $\mathbf{MA_{0.4}FA_{0.6}PbI_3}$ crystal.

We can analyze the width of electron and hole in the ODMR resonances, ΔB, defined as standard deviation of the Gaussian distribution. The width ΔB is contributed by the spread on nuclear fields Δ_{N} and by the spread of g factors Δg [2] and can be described by the equation:

$$\Delta B = \left[\Delta_{\mathrm{N}}^2/2 + \left(\frac{\Delta g}{g} B \right)^2 \right]^{1/2}. \tag{S5}$$

This equation assumes the Gaussian distributions of g factors and nuclear fields. Figure S2 summarizes ΔB, extracted from the ODMR spectra shown in Figure 2a of the main text at different rf frequencies f_{rf} in the $\mathrm{MA_{0.4}FA_{0.6}PbI_3}$ crystal at $T = 1.6$ K. Figure S2a shows the ODMR peak width ΔB of the electron and hole resonances as function of the magnetic field. The fact that ΔB weakly depends on B indicates small contribution of the g factor spread. Thus, ODMR peak width, in this range of B, is mainly determined by the nuclear field fluctuations.

Using Equation (2) in the main text, the corresponding spin dephasing time T_2^* can be evaluated. Figure S2b presents the corresponding spin dephasing times $T_{2,\mathrm{e}}^*$ and $T_{2,\mathrm{h}}^*$ as function of the magnetic field.

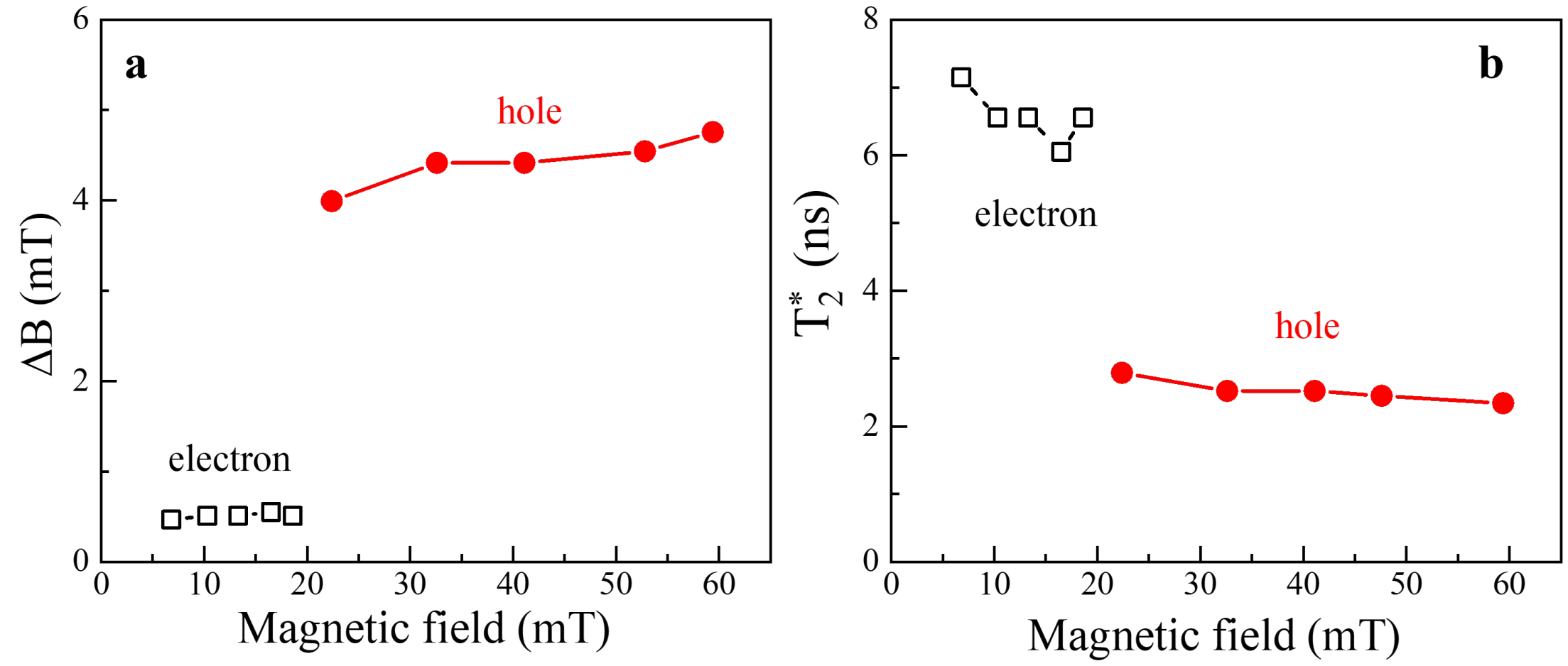

Figure S2: a) Width of the ODMR peak ΔB of the electron and hole resonances as function of the magnetic field in the $MA_{0.4}FA_{0.6}PbI_3$ crystal. b) Corresponding spin dephasing times of the electron and hole resonances as function of the magnetic field. The excitation laser energy is 1.528 eV. The laser power is 1 mW. $T = 1.6$ K.

S3. Dependence of the longitudinal spin relaxation time of electrons and holes, T_1, on laser power.

To obtain the spin relaxation time T_1 of the undisturbed spin system, we measured the ODMR signal as function of the modulation frequency for different laser powers at the fixed magnetic field strengths of $B = 31.8$ and 106 mT. Equations (S1)-(S3) provides a reasonable fit to the experimental dependencies, allowing us to extract T_1 for both electrons and holes at each laser power. The extracted $1/T_1$ values increase with increasing laser power, indicating that the optical excitation perturbs the spin system and accelerates the spin relaxation. By extrapolating the power dependence to the limit of zero laser power, we obtain the intrinsic spin relaxation times of the undisturbed system, $T_{1,\mathrm{e}} = 22$ μs for electrons and $T_{1,\mathrm{h}} = 88$ μs for holes.

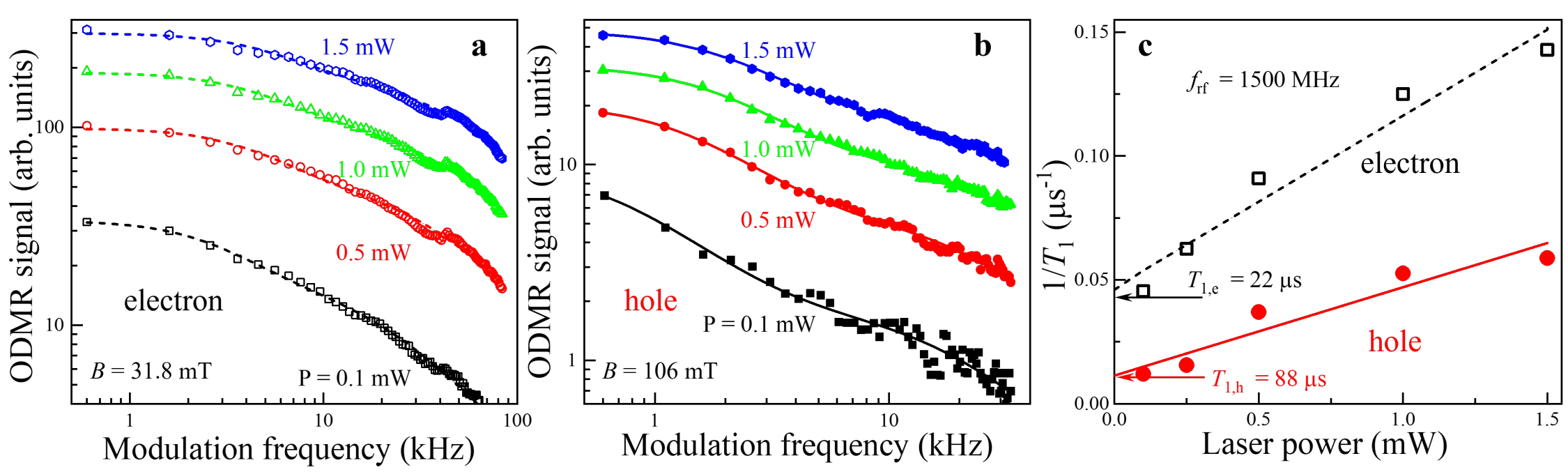

Figure S3: ODMR signal of a) electrons and b) holes in the$MA_{0.4}FA_{0.6}PbI_3$ crystal as function of the modulation frequency at $B = 31.8$ mT and 106 mT, respectively, for different laser powers. $f_{\mathrm{rf}} = 1500$ MHz. The lines show fits of the experimental data with Equations (S1-S3). c) Laser power dependence of the longitudinal spin relaxation rate $1/T_1$. The lines represent linear fits. The excitation energy is 1.528 eV. $T = 1.6$ K.

S4. Temperature dependence of the spin dephasing time T_2^*.

With increasing temperature, the spin dephasing time T_2^* decreases for both electrons and holes, as shown in Figure S4. Within a purely phenomenological description, they follow an Arrhenius-like function, Equation 5, in main text. From the fit, the activation energies for electrons and holes ($E_{\mathrm{A,e}} = 3.7$ meV and $E_{\mathrm{A,h}} = 1.3$ meV) can be obtained.

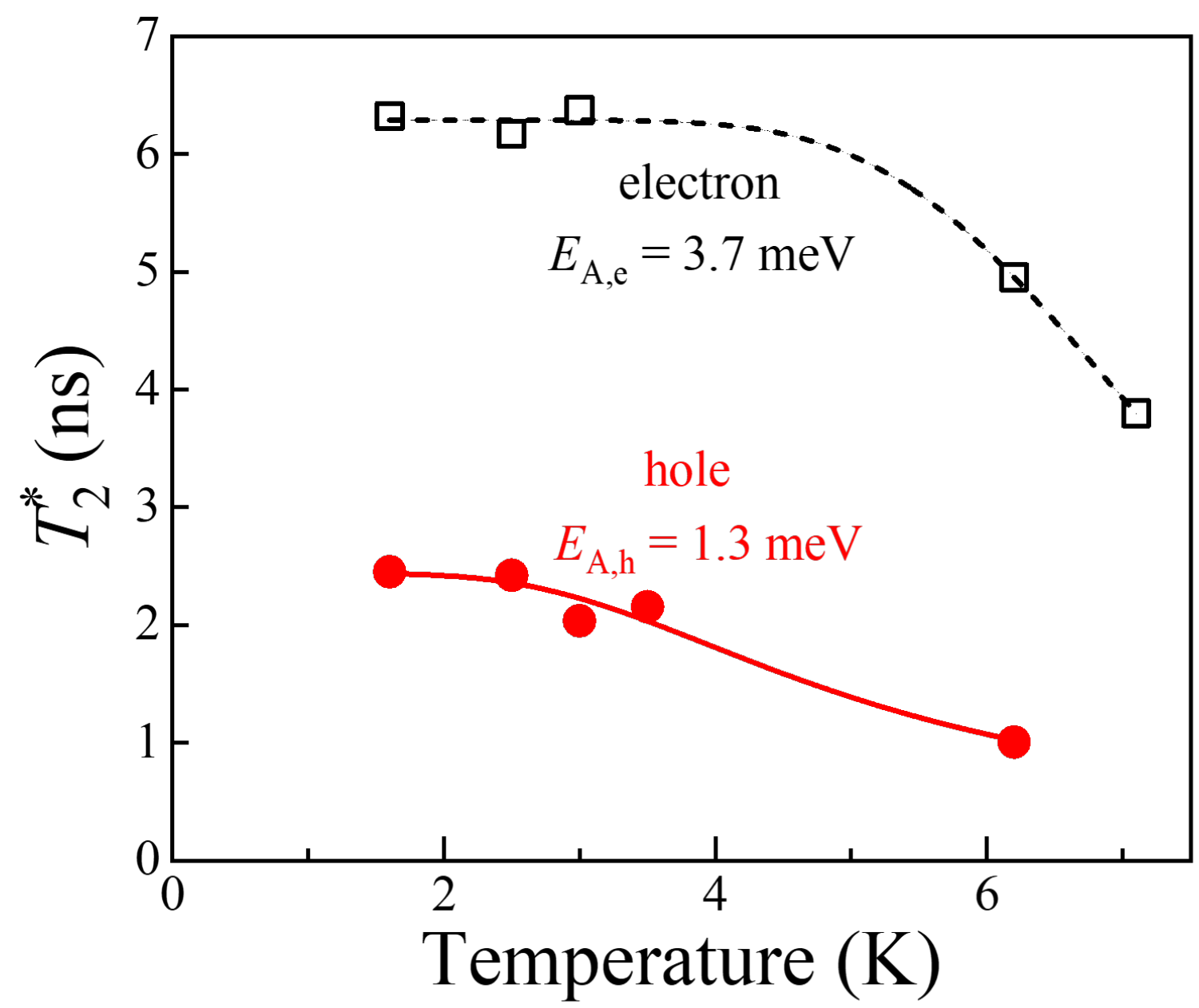

Figure S4: Temperature dependence of the spin dephasing time, T_2^*, in the $\mathrm{MA_{0.4}FA_{0.6}PbI_3}$ crystal. The lines are fits with Equation (5) in the main text. The excitation energy is 1.528 eV.

S5. ODMR signals as function of magnetic field, scanned from negative to positive direction.
Figure S5a shows the ODMR signal as function of magnetic field measured at different rf frequency on the $\mathrm{MA_{0.4}FA_{0.6}PbI_3}$ crystal at $T = 1.6$ K, using the excitation laser energy of 1.524 eV. Several resonances corresponding to different carrier subensembles are clearly resolved. The magnetic field dependence of the corresponding resonance frequencies is presented in Figure S5b, the lines show linear fits to the experimental data. Notably, the several resonances exhibit finite offsets in their frequency dependence on magnetic field. Such offsets are unlikely to originate from dynamical nuclear polarization [3], because the dependencies remain symmetric when the magnetic field direction is reversed from negative to positive values, as demonstrated in Figure S5b.

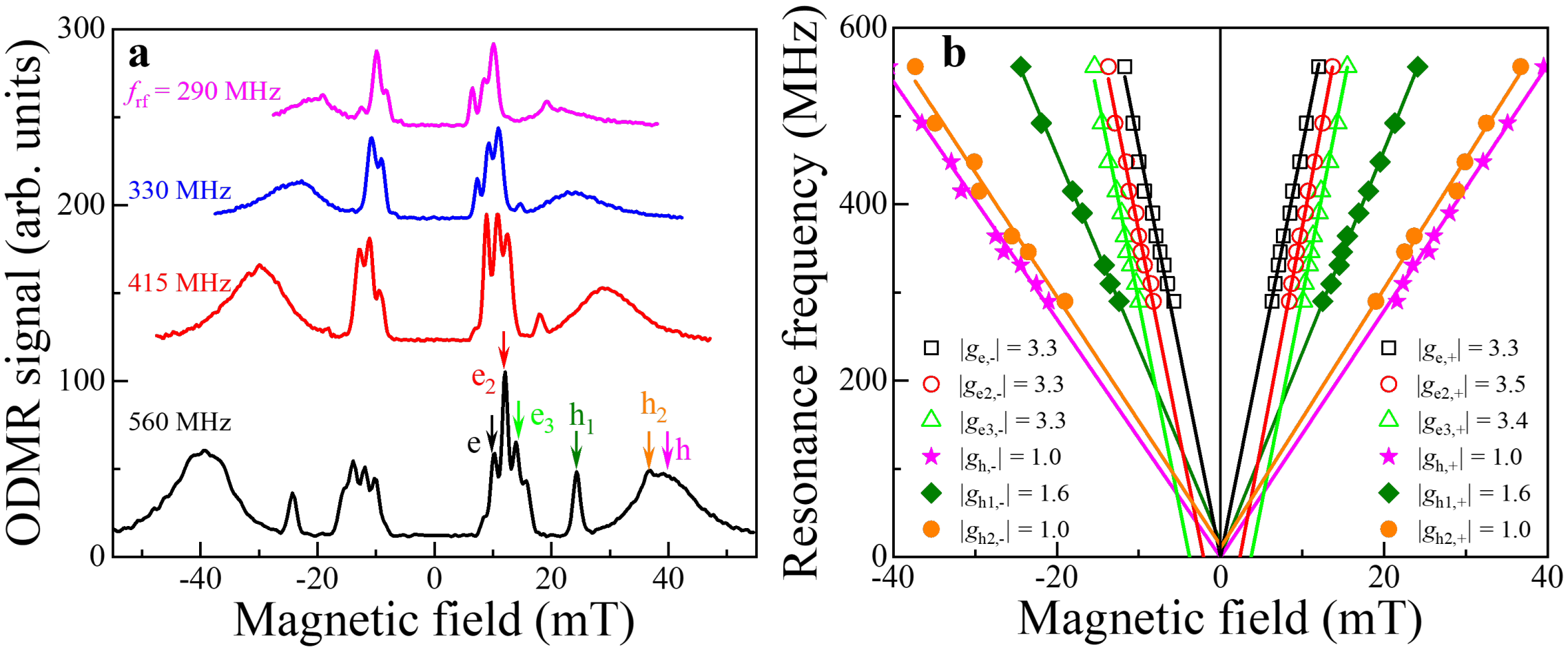

Figure S5: a) ODMR spectra of $MA_{0.4}FA_{0.6}PbI_3$ crystal measured at different rf frequencies. The curves are vertically shifted for clarity. b) Magnetic-field dependence of the resonance frequencies corresponding to electrons and holes in the ODMR spectra, with linear fits. The excitation laser energy is 1.524 eV. The laser power is 1 mW. $T = 1.6$ K.

S6. Spin dephasing time of holes as function of excitation energy.

Figure S6 shows the spin dephasing times T_2^* of the h, h_1, and h_2 resonances in the $MA_{0.4}FA_{0.6}PbI_3$ crystal as a function of the excitation laser energy. The spin dephasing time estimated from the ODMR peak width reaches $T_2^* = 17$ ns for the h_2 spin subensemble at an excitation energy of 1.522 eV.

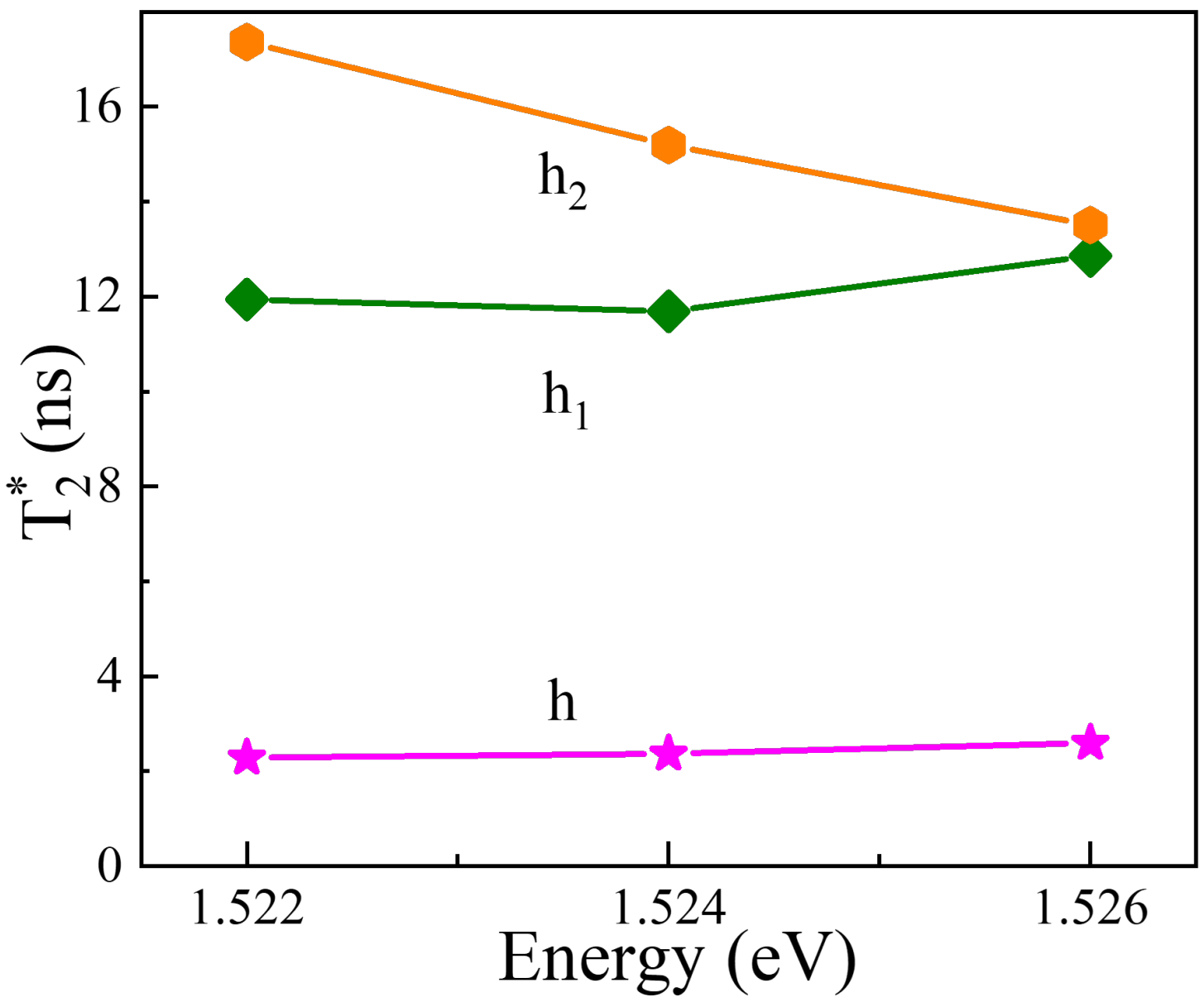

Figure S6: Spin dephasing times of the h h_1, and h_2 resonances in the $MA_{0.4}FA_{0.6}PbI_3$ crystal as function of excitation laser energy. $f_{\mathrm{rf}} = 330$ MHz. The laser power is 2 mW. $T = 1.6$ K. The magnetic fields for the h_1, h_2 and h resonances are 14.1, 21.3, and 24 mT, respectively. The lines are guides to the eye.

S7. Spin relaxation times of different electron and hole subensembles as functions of magnetic field.

Figure S7a shows ODMR signal as a function of the modulation frequency at different magnetic fields, corresponding to the electrons and holes resonances at fixed rf frequency of 560 MHz. From the fits, the spin relaxation times for electrons and holes can be obtained. The resulting spin relaxation times for electrons and holes are plotted as a function of magnetic field in Figure S7b.

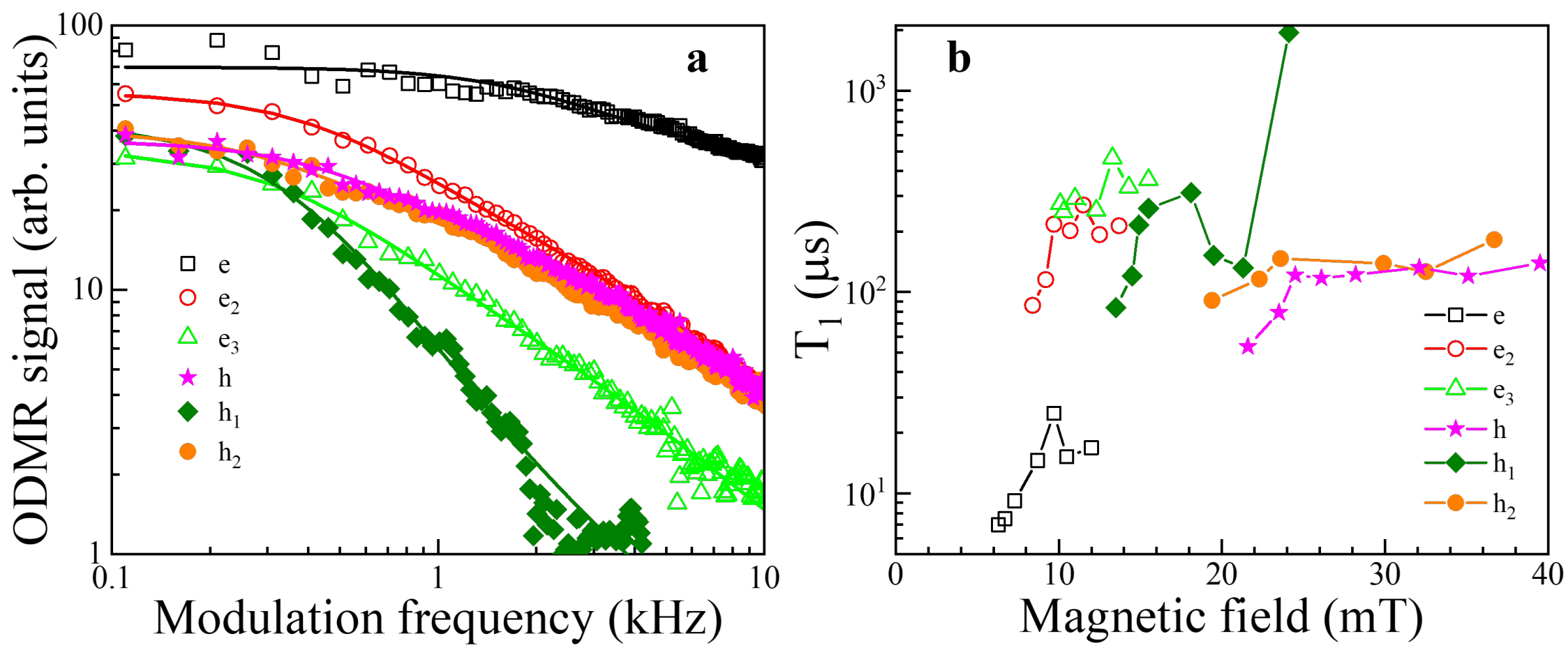

Figure S7: a) ODMR signals of $MA_{0.4}FA_{0.6}PbI_3$ crystal as a function of the modulation frequency at different magnetic fields, corresponding to the electron and hole resonances, at the fixed rf frequency of 560 MHz. The lines show fits of the experimental data with Equations (S1)-(S4). b) Spin relaxation times for different electron and hole subensembles as a function of the magnetic field. The excitation laser energy is 1.524 eV. The laser power is 1 mW. $T = 1.6$ K.

.

S8. ODMR investigation in $MA_{0.8}FA_{0.2}PbI_3$ single crystal.

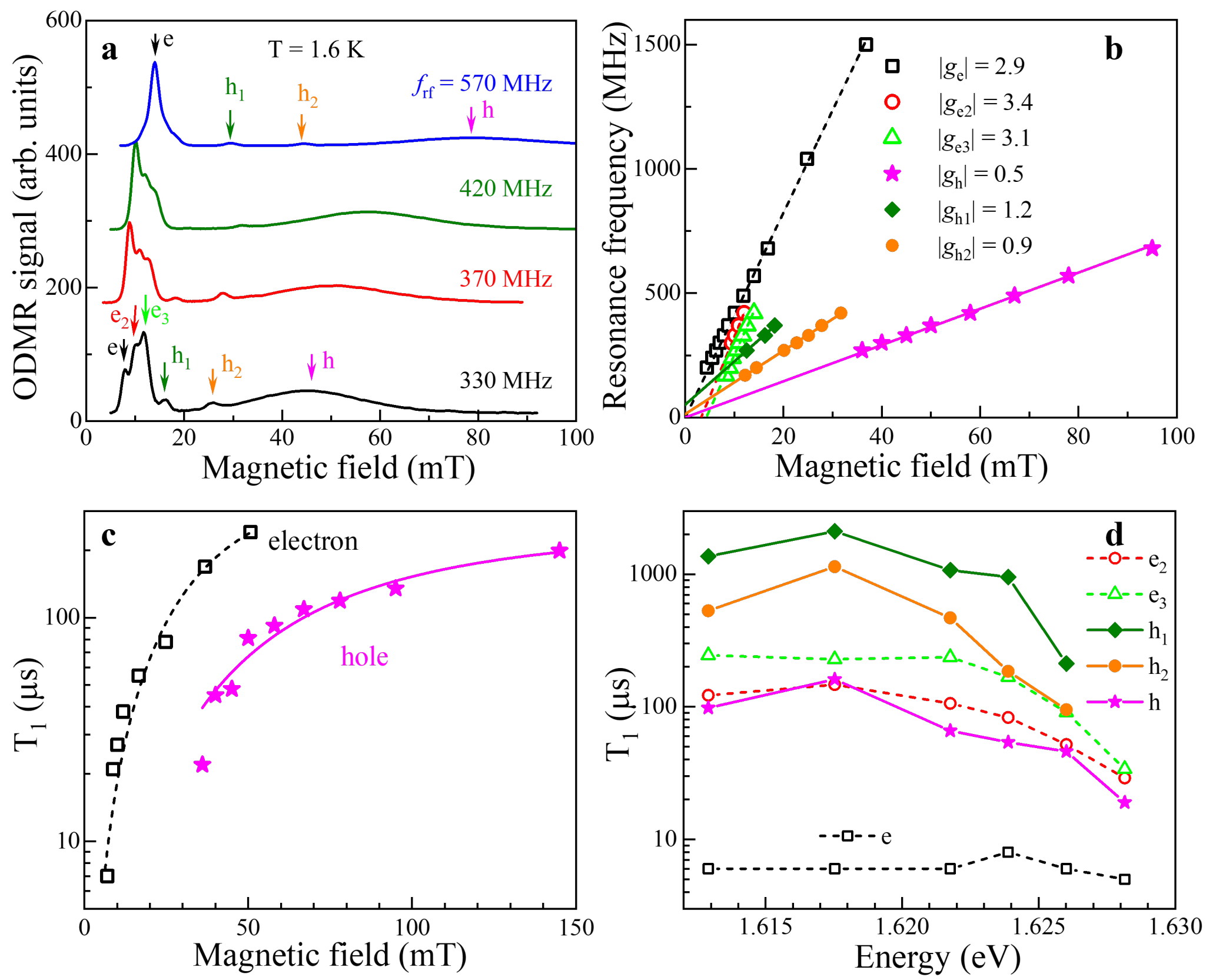

Figure S8: a) ODMR spectra of the $MA_{0.8}FA_{0.2}PbI_3$ crystal measured at different rf frequencies. The curves are vertically shifted for clarity. b) Magnetic-field dependence of the resonance frequencies corresponding to the electron and hole positions in the ODMR spectra, with the associated linear fits shown by the solid lines. c) Spin relaxation times T_1, for electrons and holes as function of magnetic field. The lines show fits of the experimental data using Equation (4) in the main text. d) T_1 as function of the laser photon energy for different subensembles of electrons and holes with the fixed rf frequency of 330 MHz. The laser power is 2 mW, $T = 1.6$ K. The laser energy in (a),(b),(c) is 1.626 eV.

Multiple carrier resonances are also found for the $MA_{0.8}FA_{0.2}PbI_3$ crystal as shown in Figure S8. With decreasing rf frequency, the number of peaks in the ODMR spectra increases. We determine the g-factors, corresponding to the different ODMR peaks from the slope of the linear fit of the resonance frequency dependence on the magnetic field shown in Figure S8b. We assign the observed spin components to distinct carrier subensembles: the electron subensembles e, e_2, and e_3 with g-factors of 2.9, 3.4, and 3.1, respectively, and the hole subensembles h, h_1, and h_2 with g-factors of 0.5, 1.2, and 0.9, respectively. The energy dependence of T_1 for different subensembles in this sample is shown in Figure S8d. A decrease of the laser photon energy leads to an increase of the spin relaxation times for all carrier spin subensembles, similar to the tendency found in the $MA_{0.4}FA_{0.6}PbI_3$ crystal (Figure 4d in the main text). For the h_1 hole spin subensemble, T_1 can reach 2 ms at the laser energy of 1.617 eV. The other carrier subensembles exhibit faster relaxation, reflecting varying degrees of localization and different Overhauser fields.

References

[1] V. V. Belykh, S. R. Melyakov, *Phys. Rev. B* **2022**, *105*, 20 205129.

[2] V. V. Belykh, M. M. Glazov, S. R. Meliakov, D. R. Yakovlev, E. V. Kulebyakina, M. L. Skorikov, M. V. Kochiev, M. S. Kuznetsova, E. V. Kolobkova, M. Bayer, *arXiv preprint arXiv:2603.03038* **2026**.

[3] E. Kirstein, D. R. Yakovlev, M. M. Glazov, E. Evers, E. A. Zhukov, V. V. Belykh, N. E. Kopteva, D. Kudlacik, O. Nazarenko, D. N. Dirin, M. V. Kovalenko, M. Bayer, *Adv. Mater.* **2022**, *34*, 1 2105263.
